# Presumptive Cycad Toxicosis in a Dog. Clinical and Magnetic Resonance Imaging Findings: A Case Report

**DOI:** 10.3389/fvets.2020.00468

**Published:** 2020-08-14

**Authors:** Christian Maeso, Carles Morales, Rafael Obrador, Eva Abarca, Inés Carrera

**Affiliations:** ^1^Neurology Department, Ars Veterinaria Hospital, Barcelona, Spain; ^2^Critical Care Department, Ars Veterinaria Hospital, Barcelona, Spain; ^3^Ophthalmology Department, Ars Veterinaria Hospital, Barcelona, Spain; ^4^Diagnostic Imaging Department, Willows Veterinary Hospital, West Midlands, United Kingdom

**Keywords:** case report, cycad, *Cycad revoluta*, MRI, toxic encephalopathy, toxicosis, white matter

## Abstract

Cases of cycad toxicosis have been described in dogs that have presented with gastrointestinal, hematologic, hepatic, neurological, and carcinogenic signs. This case report describes brain magnetic resonance imaging (MRI) lesions in a dog with gastrointestinal and neurological signs secondary to cycad toxicosis. A 5-year-old neutered female Jack Russell terrier presented with a 2-days history of gastroenteric signs, progressive generalized tremors, and altered mentation after possible ingestion of *Cycad revoluta*. Neurologic examinations revealed disorientation, a wide-based stance, severe spasticity of the four limbs, intention tremors, severe cerebellar ataxia, decreased postural reactions in all four limbs, and intermittent decreased menace response in both eyes—all of which are consistent with a multifocal intracranial disorder involving the forebrain and cerebellum. A brain MRI showed diffuse/ill-defined, intra-axial bilateral and symmetrical changes, predominantly affecting the white matter of the cerebral hemispheres, thalamus, hippocampus, and cerebellum. A presumptive diagnosis of toxic-metabolic encephalopathy was made. Medical management of the clinical signs was performed, and the dog was discharged 7 days after presentation with no neurological abnormalities. Two and 8 weeks later, complete blood count (CBC), chemistry, electrolytes, and 8 weeks later brain MRI were performed, revealing no abnormalities. To the best of the authors' knowledge, this is the first case report describing lesions detected by brain MRI secondary to cycad toxicosis as well as a complete resolution of brain lesions on a follow-up MRI 8 weeks later.

## Background

Cycad plants, also known as sago palms, can be found throughout the globe, particularly in tropical and subtropical regions, as native or ornamental plants. Cycad toxicosis has been described in different species, including sheep, cattle, horses, pigs, and dogs as well as in humans (1–8). Although all parts of the plant can be toxic, the seeds and roots seem to contain the highest concentrations of toxins (9). Three types of toxins are recognized in cycad plants: azoxyglycosides (cycasin), β-methylamino-L-alanine (BMAA), and an unidentified compound with a high molecular weight (10). Methylazoxymethanol (MAM) is produced in the gastrointestinal tract as an active metabolite from cycasin with multiple toxic properties, including hepatotoxic and neurotoxic characteristics (1, 10, 11). BMAA is an *N*-methyl-d-aspartate (NMDA) agonist, which has a similar effect to glutamate, producing a cascade of enzyme activation leading to neuronal death. Therefore, BMAA appears to play a role in the development of neurological clinical signs (1, 12). The unidentified high-molecular-weight compound has been associated with hindlimb paralysis and axonal degeneration in intoxicated cattle (2, 4)

Descriptions of cycad toxicosis in dogs presenting with gastrointestinal, hematologic, hepatic, neurological, and carcinogenic signs are limited to a few case reports and three retrospective studies (3, 6–8, 13, 14). None of these studies includes magnetic resonance imaging (MRI) studies. This case report describes presumptive cycad toxicosis in a dog with an acute onset of gastrointestinal and neurological signs, who underwent brain MRI at the time of the presentation and again at follow-up 8 weeks later.

## Case Presentation

A 5-year-old, neutered, female Jack Russell terrier was referred to the neurology service of Ars Veterinaria Hospital for acute vomiting, progressive generalized tremors, and altered mentation. The dog had no prior history of systemic and/or neurological problems and was up to date on vaccinations and deworming regimes. She lived without other animals in a house with a garden.

Two days before referral, the owners witnessed the dog eating *Cycad revoluta* seeds. Two hours after ingesting the seeds, the dog vomited seven times in <3 h. The first-opinion veterinarian performed an abdominal radiograph, which was unremarkable, and prescribed a therapy of a single dose of amoxicillin/clavulanic acid, maropitant, ranitidine, and a B complex.

The day before the referral and despite the treatment, the dog exhibited clinical worsening with generalized weakness and tremors, incoordination, and ptyalism. A second veterinary assessment identified tachycardia (200 bpm), mild dehydration (6%), altered mentation, ataxia of all four limbs, generalized tremors, and bilateral mydriasis. A complete blood count (CBC), serum biochemistry, ammonia levels on serum, coagulation test [prothrombin time (PT), partial thromboplastin time (aPTT)], and a urinalysis were performed, showing only a mild elevation of ALT 131 U/L (reference range 10–125 U/L), and an aPTT of 123 s (reference range 72–102 s). Thoracic radiographs and an abdominal ultrasound were unremarkable. A treatment regime consisting of fluid therapy with a dose of 1 mg/kg subcutaneous (SC) maropitant, a midazolam constant rate infusion (CRI) at 0.2 mg/kg/h intravenously (IV) and 1 g/kg oral (PO) activated charcoal was established. However, the clinical signs continued to worsen, and the dog was referred to our institution 24 h later.

On presentation, a physical examination only indicated abdominal pain. Arterial blood pressure measured by the oscillometric method showed a systolic arterial pressure of 145 mm Hg (normal range <140 mm Hg). A neurological examination was performed, which revealed disorientation, a wide-based stance, severe spasticity of the four limbs, intention tremors, severe cerebellar ataxia with an inability to maintain balance due to the cerebellar dysfunction, decreased postural reactions in all four limbs, and an intermittent decreased menace response in both eyes. The neuroanatomic localization was intracranial with involvement of the forebrain and cerebellum. Based on the clinical history and the physical and neurological findings, the differential diagnosis list included toxic, metabolic, and—although less likely—inflammatory/infectious diseases. Therefore, a CBC, serum biochemistry (including bile acids and serum ammonia), electrolytes, a brain MRI, and cerebrospinal fluid (CSF) collection were planned.

Bile acids and serum ammonia were within normal limits. There was a mild increase in the ALT activity 153 U/L (reference range 10–125 U/L).

The dog underwent general anesthesia, and an MRI of the brain was performed using a high-field 1.5 Tesla scanner (Vantage Elan, Canon Medical Systems). The sequences acquired included T2-weighted (T2W) fast spin-echo (FSE) images in the dorsal (TR: 5,952 ms/TE: 105 ms), sagittal (TR: 4,652 ms/TE: 105 ms), and transverse (TR: 6,760 ms/TE: 105 ms) planes. Additional sequences included fluid-attenuated inversion recovery (FLAIR) (TR: 8,000 ms/TE: 120 ms), T2^*^ gradient echo (TR: 870 ms/TE: 15.0 ms), and pre- and post-contrast sequences with Gadoteridol at a dosage of 0.1 mmol/kg IV of body weight with T1-weighted (T1W) FSE (TR: 479 ms/TE: 11.0 ms) images in the transverse plane.

**Figure 1:**
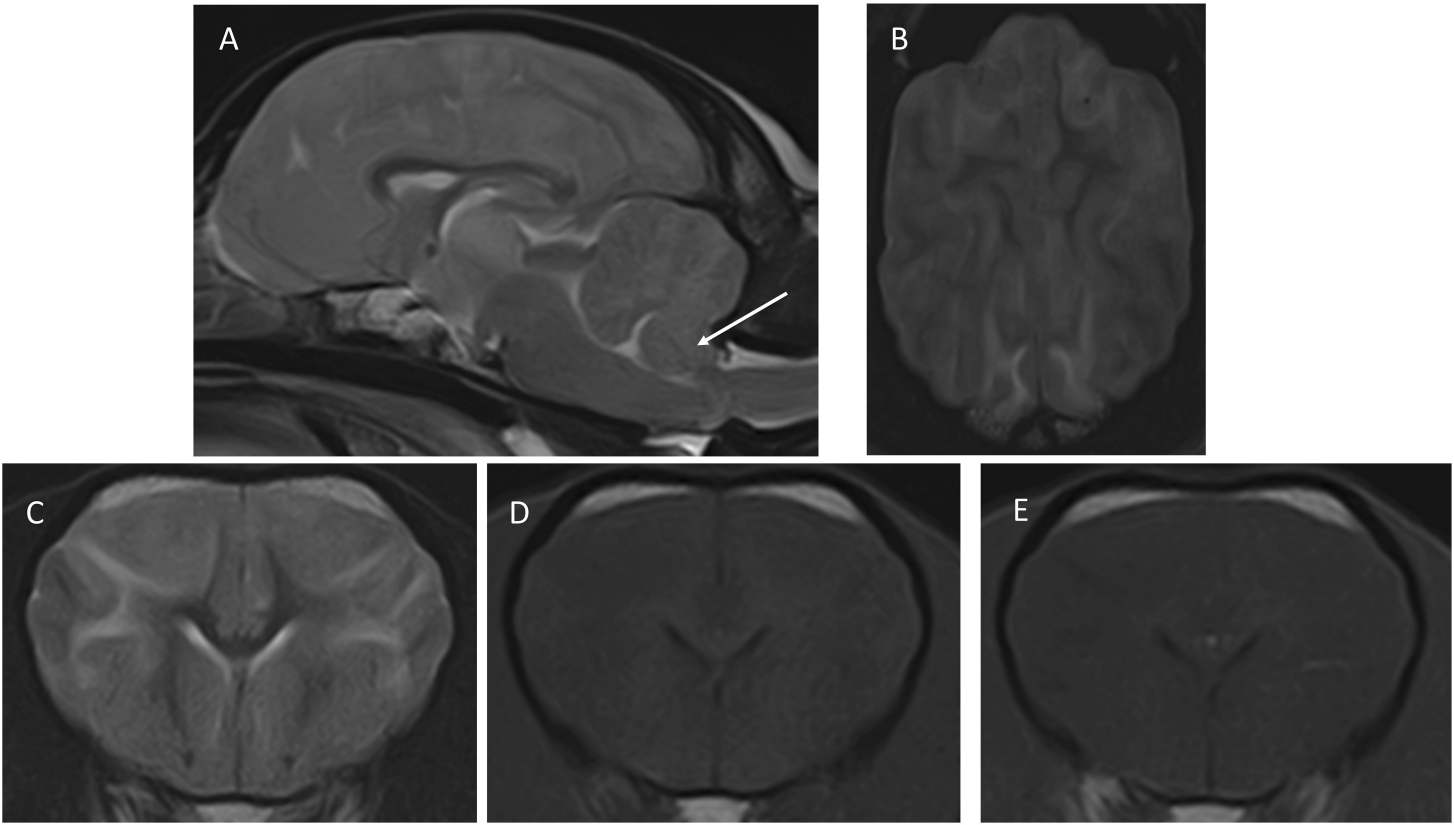
Images of the first MRI study performed at presentation. **(A)** Mid-sagittal T2W, **(B)** dorsal T2W, **(C)** transverse, T2W, **(D)** T1W pre-contrast, **(E)** transverse T1W post-contrast. The transverse images are at the level of the nucleus caudatus, and the dorsal image is a slice taken just dorsal to the corpus callosum. The arrow in **(A)** points to the caudo-ventral aspect of the cerebellum, which is displaced caudally and is partially herniating through the foramen magnum. In **(B)**, notice the blurring of the cerebral sulci, indicating increased brain volume, and the diffuse increased signal intensity of the white matter, which is also seen in **(C)**. The signal intensity changes of white matter are barely perceptible in T1W **(D,E)** shows a lack of contrast enhancement.

The MRI revealed bilateral and symmetrical intra-axial and diffuse/ill-defined lesions predominantly affecting white matter, extending from the frontal to the occipital area (Figure 1). The corona radiata and occipital lobes were markedly affected. In addition, there were ill-defined lesions affecting the thalamus bilaterally (slightly asymmetric and more pronounced on the right side) as well as the hippocampus (bilateral and diffuse changes) and cerebellar white matter. Lesions were homogeneous in all sequences, hyperintense in T2W and FLAIR, and iso- to hypointense in T1W in comparison to white matter without evidence of contrast enhancement. The T2^*^ gradient echo sequence did not reveal the presence of hemorrhage. The combination of these intra-axial lesions caused a blurring of the cerebral and cerebellar sulci and gyri of the frontal, parietal, and occipital cortex. In addition, the subdural space was not clearly visualized, and the lateral ventricles showed a flattened, slightly distorted shape in their caudal part, due to an increase in the volume of the hippocampus. The caudo-ventral aspect of the cerebellum was displaced caudally, partially herniating through the foramen magnum. All these findings were indicative of brain swelling (secondary to generalized brain oedema) and cerebellar herniation—which together were suggestive of increased intracranial pressure (ICP). The main differential diagnosis—taking the dog's clinical history into account—was metabolic or toxic encephalopathy. The presence of inflammatory/infectious encephalopathy could not be completely ruled out from these images, but it was considered less likely.

CSF collection was not performed because of the presence of signs suggestive of increased ICP. Immediately after the MRI study, mannitol CRI (0.5 g/kg over 20 min IV) and methylprednisolone (1 mg/kg IV) were administered. The dog was hospitalized, and therapy with maropitant (1 mg/kg SID IV), omeprazole (1 mg/kg BID IV), methylprednisolone (0.5 mg/kg SID IV), and activated charcoal (1 g/kg TID PO) as well as fluid therapy (50 ml/kg/24 h IV CRI plus replacement of a dehydration deficit of 5% body weight for the first 24 h, followed by a maintenance rate after that), were administered. We established a guarded prognosis.

Two days after admission, the neurological and systemic status of the dog remained unchanged.

On day 3 postadmission, neurological signs improved slightly with the dog displaying moderate cerebellar ataxia and mild intention tremors and the ability to maintain balance. Additionally, the dog showed severe hypertension with a systolic arterial pressure varying between 170 and 230 mm Hg, macroscopic hematuria, and abdominal petechiae. With the aim of diagnosing a possible coagulation disorder, CBC, biochemistry, and coagulation tests (PT, aPTT) were repeated and showed severe thrombocytopenia of 30 × 10^9^/L (reference range 148–484 × 10^9^/L) and an aPTT of 135 s (reference range 72–102 s). Hepatic parameters, including ALT enzymatic activity, were within the normal range. A Snap 4DX (*Dirofilaria immitis, Borrelia burgdorferi, Ehrlichia canis, Ehrlichia ewingii, Anaplasma phagocytophilum, Anaplasma platys*) test was negative, and a fast abdominal and thoracic ultrasound examination showed a mild amount of free abdominal fluid. A complete ophthalmic examination was performed, which revealed extensive subconjunctival hemorrhaging in both eyes as well as punctate hemorrhages on the surface of the iris. Funduscopic examination showed tortuous retinal vessels with a focal area of bullous retinal detachment (twice the size and adjacent to the lateral portion of the optic nerve head in the left eye). Additional treatments were amlodipine (0.1 mg/kg BID PO) administered to treat systemic hypertension, N-acetylcysteine (70 mg/kg QID IV) administered as a hepatoprotective, and vitamin K_1_ (5 mg/kg SID SC).

On day 4 postadmission, the dog had improved considerably, showing only moderate, intermittent hypertension with systolic blood pressure between 140 and 170 mmHg and diarrhea, in which seeds could be observed. This finding supported the suspicion that the dog had ingested *Cycad revoluta*. A funduscopic examination revealed a dramatic improvement in the focal bullous retinal detachment.

On day 7 postadmission, neurologic and intraocular examinations were unremarkable. There were no signs of arterial hypertension, and the subconjunctival and iris hemorrhages had improved markedly. Only moderate diarrhea was ongoing. The platelet count was 140 × 10^9^/L (reference range 148–484 × 10^9^/L). The dog was discharged with metronidazole (12.5 mg/kg BID for 7 days), prednisolone (0.5 mg/kg SID) for 3 more days with a tapering schedule of 5 days, omeprazole (1 mg/kg BID for 3 weeks), amlodipine (0.15 mg/kg SID), and s-adenosylmethionine (80 mg/dog SID).

**Figure 2:**
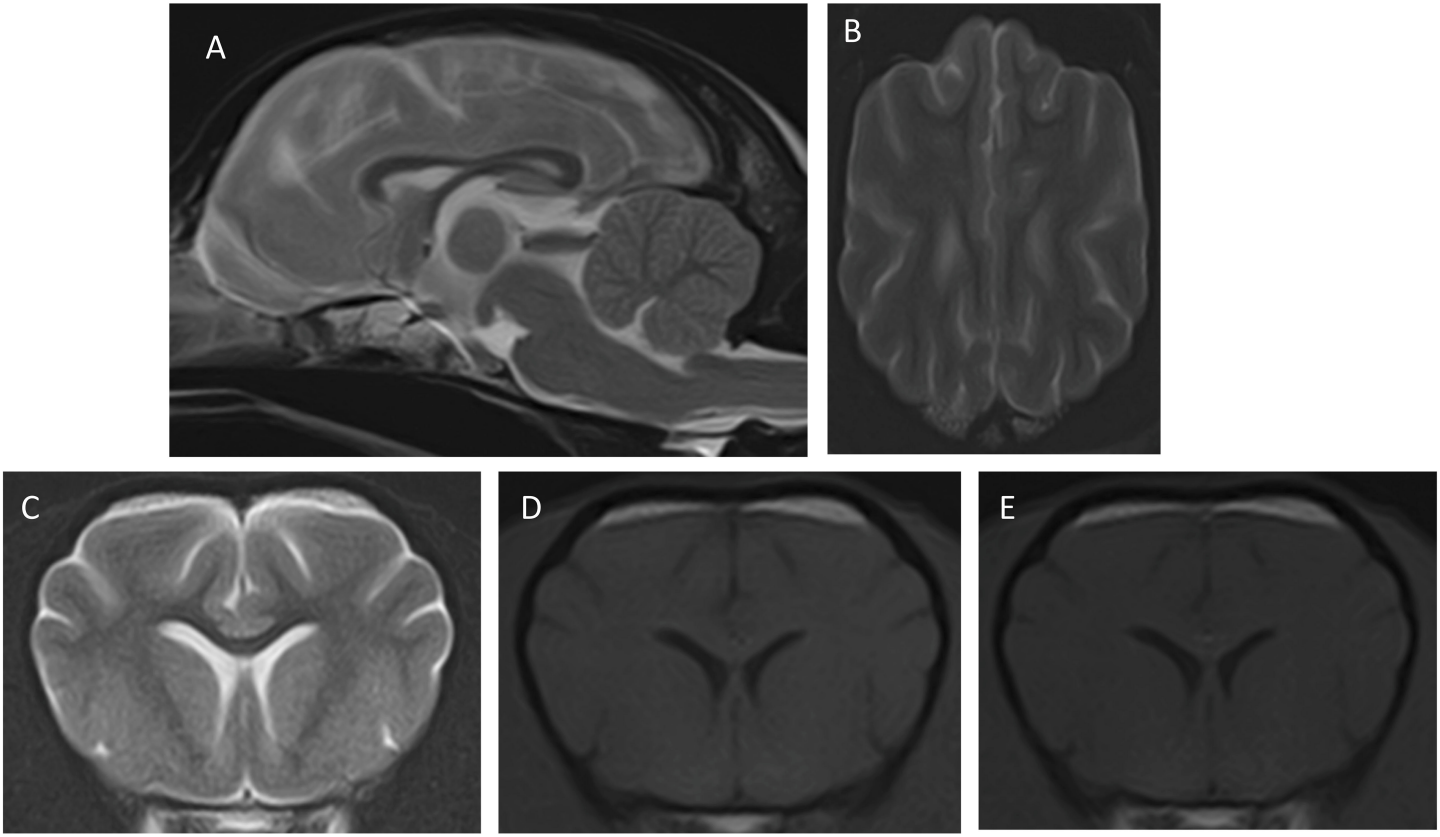
Images of the second MRI study after the resolution of the clinical signs. The transverse and dorsal images are at similar levels as those in Figure 1. **(A)** Mid-sagittal T2W, **(B)** dorsal T2W, **(C)** transverse, T2W, **(D)** T1W pre-contrast, **(E)** transverse T1W post-contrast. Notice that the cerebellum shows normal position **(A)**, the cerebral sulci are well-delineated **(B)**, and the white and gray matter show good definition with normal signal intensity in all sequences.

Follow-up evaluations were carried out 1 week and 8 weeks after discharge. Physical, neurological, and ophthalmologic examinations showed no abnormalities, and amlodipine was discontinued due to the absence of systemic hypertension. CBC, biochemistry, and electrolytes were performed in both follow-up evaluations, which were unremarkable. A brain MRI was performed at the 8-weeks follow-up, revealing a complete resolution of the changes described previously (Figure 2). In a follow-up phone call with the owners, 8 months after discharge, the dog was reported to be clinically healthy.

## Discussion

Cycad toxicosis has been reported in dogs before and has shown high morbidity and mortality rates (6–8). To the best of the authors' knowledge, this is the first report describing the brain MRI features in a dog with presumed cycad intoxication with clinical resolution after appropriate treatment and resolution of the brain lesions proven by a follow-up MRI after 8 weeks.

Cycad toxicities have been described as chronic in humans and cattle (2, 15, 16) and as acute in dogs (6). Clinical signs of toxicity in canine patients have been reported within 4 h of ingesting *Cycad revoluta* seeds and include gastrointestinal, liver, and neurological abnormalities (1, 6–9, 13, 14).

The main toxin responsible for the gastrointestinal signs and hepatotoxicity is believed to be the MAM compound, which is metabolized from the intestinal flora. Reported gastrointestinal clinical signs in intoxicated dogs include vomiting, diarrhea, melena, hematochezia, decreased appetite, and ptyalism (7, 8, 10). In accordance with these reports, the dog in this case study had acute gastrointestinal signs characterized by vomiting and ptyalism within 4 h of cycad ingestion.

Neurological signs are described in dogs with cycad toxicosis and are characterized by tremors, ataxia, obtundation, seizures, head pressing, and conscious proprioception deficits (3, 6–8, 13, 14). The neurological deficits of the dog in this case report were characterized by disorientation, cerebellar ataxia, and decreased postural reactions in all four limbs as well as a wide-based stance, severe rigidity of the four limbs, intention tremors, and intermittent decreased menace response in both eyes. No cerebellar signs have been described in previous studies of dogs with cycad intoxication (3, 6–8, 13, 14).

An MRI study of the dog's brain revealed bilateral and symmetric intra-axial and diffuse/ill-defined lesions predominantly affecting the white matter of the cerebrum and cerebellum, with increased brain volume and cerebellar herniation. These MRI findings were most compatible with diffuse brain edema and correlated with the neurological clinical signs, including the cerebellar ones.

The exact pathophysiology of CNS damage by cycad intoxication is not yet understood. One hypothesis is that the toxin BMAA mimics the effect of glutamate by overstimulating the NMDA receptors, resulting in a cascade of enzyme activation leading to neural damage due to excitotoxicity and, ultimately, cell death (17–19). The MRI findings reported in this case study were suggestive of diffuse brain disease and could, at least partially, correlate with this theory. However, no histopathology of the brain was available as the dog survived.

There is only one case report describing the histopathological findings of the CNS in a dog with cycad intoxication (14), which described lesions affecting the cerebellum that were characterized by mild nerve fiber degeneration in the cranial cerebellar peduncle. Specific lesions affecting the cerebellar peduncles were not shown in the MRI sequences in our dog. In cattle (2, 4), the unidentified high-molecular-weight compound is suspected of causing demyelination and axonal degeneration of the brain, spinal cord, and dorsal root ganglia. However, neurological signs in cattle are reported as part of a chronic phase in contrast with an acute phase in canine patients.

Given the acute onset of clinical signs and the predominant white matter distribution seen from the MRI images, this case may resemble acute metabolic leukoencephalopathies, which are reported in human medicine as posterior reversible encephalopathy syndrome (PRES). This syndrome is characterized by reversible subcortical vasogenic edema in patients with acute neurological signs (20, 21). The exact pathophysiology of PRES is unknown but includes two main theories: (1) hypertensive acute changes in blood pressure that lead to loss of autoregulation in cerebral circulation causing hyperperfusion, endothelial damage, and vasogenic edema or (2) cytotoxic endothelial dysfunction caused by circulating endogenous or exogenous toxins, leading to vasoconstriction, hypoperfusion, and subsequent cerebral ischemia and vasogenic edema (20, 21). Hypertension appears unlikely in this case because the dog was not showing systemic hypertension either previously or at the time of the MRI study. However, arterial hypertension developed in this dog from day 3 with a systolic arterial pressure range of between 170 and 230 mm Hg (mean 200 mm Hg).

Another relevant finding of this case was the presence of subconjunctival and iridal hemorrhages as well as an increase in the retinal vessel's tortuosity and retinal bullous detachment. These findings have not been described in previous studies of cycad intoxication. We hypothesize that these ocular changes could be related to the acute and severe systemic hypertension that developed over the course of the disease (as explained above), but it is also possible that thrombocytopenia contributed to the petechiae and hemorrhages.

The treatment of cycad plant ingestion in dogs focuses on decontamination to prevent/limit toxin absorption and supportive care for dehydration, liver disease, hypocoagulability, and/or DIC (1, 7–9). In this case, the dog arrived 48 h after onset of initial clinical signs that included vomiting, so decontamination was limited to the administration of activated charcoal. Activated charcoal has been shown to have a protective effect in dogs with cycad toxicosis, mainly due to the reduction in the gastrointestinal absorption of toxins (7) as the amount of toxin absorbed relative to the dog's weight determines the severity of the liver injury. Next, the dog received supportive treatment for dehydration, liver support (N-acetylcysteine and vitamin K_1_), and systemic hypertension (amlodipine). A study in people (22) demonstrated the effectiveness of NMDA receptor antagonist MK801 in the treatment of cycad toxicosis. Therefore, it is important to highlight the need for more descriptions related to specific treatments in cycad toxicosis in dogs.

Two retrospective studies (7, 8) evaluated the outcome of cycad intoxication in dogs with a variable survival rate of between 36 (8) and 50% (7) Interestingly, nadir serum albumin levels and/or nadir platelet counts have been described as predictors for the outcome (7, 8), in which non-survivors had significantly lower values compared to survivors. However, our dog had a good outcome despite the presence of severe thrombocytopenia.

Reported clinical signs attributed to liver disease include icterus and petechiae (7, 8). The MAM molecule is metabolized in the large bowel, and once absorbed, this toxin has an enterohepatic circulation that increases exposure to the toxin (10). MAM may cause acute hepatic failure due to centrilobular necrosis, periportal fibrosis, and bile duct proliferation (7, 8). Clinical pathological abnormalities common in acute cycad toxicosis include hyperbilirubinemia, and increased ALT and alkaline phosphatase activities as well as hypocholesterolemia, hypoalbuminemia, and either hypoglycemia or hyperglycemia. Coagulation abnormalities have been commonly reported with 50% of dogs showing elevations in aPTT and approximately 33% showing thrombocytopenia (6–8). The dog in our case study showed a mild elevation of ALT and aPTT and marked thrombocytopenia, which could also indicate DIC; however, no other abnormalities were noted and hepatic enzyme activities normalized. This is interesting to note as some previously reported dogs went through a chronic phase of cycad toxicity, secondary to liver damage (8). A limitation in our case is that we did not follow-up with liver function tests excluding chronic hepatic disease; however, the dog remained clinically unremarkable after 8 weeks and on phone follow-up after 9 months.

There are some limitations to this case report. First, a toxicological analysis of the seeds present in the feces could not be performed. However, the owners judged the ingestion of cycad seeds to be a given, which was confirmed by the gross examination of feces and the correlation with the clinical signs. The second limitation was the lack of a CSF examination and that complete liver function tests were not done. CSF collection was not performed because there were signs of cerebellar herniation from the MRI, and CSF collection was not, therefore, deemed to be safe. Another limitation was the lack of DWI and ADC map sequences to distinguish cytotoxic from vasogenic edema. Further studies of cycad intoxication, including these MRI sequences, will be useful to understand the pathophysiology of this intoxication in more depth.

## Concluding Remarks

In conclusion, cycad toxicosis should be suspected in dogs that have access to cycad plants and that present with gastrointestinal, hepatic, and intracranial neurological signs. In this context, brain MRI is recommended as an important diagnostic step to assess the severity of brain damage and provide information for adequate treatment.

## Data Availability Statement

The original contributions presented in the study are included in the article/supplementary material, further inquiries can be directed to the corresponding author/s.

## Ethics Statement

Written informed consent was obtained from the owners for the participation of their animals in this study.

## Author Contributions

CMa, CMo, and IC: conception and design and editing and reviewing the draft. CMa and CMo: acquisition of data. All authors participated in the review of the manuscript.

## Conflict of Interest

The authors declare that the research was conducted in the absence of any commercial or financial relationships that could be construed as a potential conflict of interest.
